# Effects of formic acid and glycerol monolaurate on weanling pig growth performance, fecal consistency, fecal microbiota, and serum immunity

**DOI:** 10.1093/tas/txac145

**Published:** 2022-10-22

**Authors:** Payton L Dahmer, Olivia L Harrison, Cassandra K Jones

**Affiliations:** Department of Animal Sciences and Industry, Kansas State University, Manhattan, KS, USA; Department of Animal Sciences and Industry, Kansas State University, Manhattan, KS, USA; Department of Animal Sciences and Industry, Kansas State University, Manhattan, KS, USA

**Keywords:** acidifiers, fecal microbiota, growth performance, immune, nursery pig

## Abstract

A total of 350 weanling pigs (DNA 400 × 200; initially, 5.67 ± 0.06 kg BW) were used in a 42-day study with 5 pigs per pen and 14 replicate pens per treatment. At weaning, pigs were allotted to pens in a completely randomized design and pens of pigs were randomly assigned to one of five dietary treatments: 1) negative control (**CON**; standard nursery diet containing only 150 ppm Zn from trace mineral premix and no acidifier); 2) control diet with 3,000 ppm added zinc from ZnO included in phase 1 and 2,000 ppm added zinc from ZnO included in phase 2 **(ZnO)**; 3) control diet with 0.70% formic acid (**FA**; Amasil NA; BASF, Florham, NJ); 4) control diet with 0.18% glycerol monolaurate (**GML**; Natural Biologics GML, Natural Biologics, Newfield, NY); and 5) control diet with a 1.0% blend of formic acid and glycerol monolaurate (**FORMI**; FORMI 3G, ADDCON GmbH, Bitterfeld-Wolfen, Germany). Pigs were fed treatment diets from d 0 to d 28 and were then fed a common diet from d 28 to d 42. From days 0 to 7, pigs fed ZnO or FORMI had increased (*P* = 0.03) ADG compared to pigs fed CON, with no difference in feed intake (*P* > 0.05). Overall, pigs fed GML had reduced (*P* < 0.0001) ADG compared with those fed the CON, ZnO, or FORMI diets. Fecal DM was evaluated from days 7 to 28 and there was a treatment × day interaction (*P* = 0.04). Pigs fed GML had a lower fecal DM % on day 7, but a higher fecal DM % on days 14 and 21; however, no differences in fecal DM were observed on day 28. Fresh fecal samples were collected from the same randomly selected pig on days 0 and 14 (70 pigs total;14 pigs per treatment) for analysis of fecal microbial populations using 16S rDNA sequencing. Dietary treatment did not significantly impact fecal microbiota at the phyla level, but pigs fed ZnO had an increased relative abundance (*P* < 0.01) of the family *Clostridiaceae*. A blood sample was also collected from one pig per pen on days 0 and 14 for analysis of serum IgA, IgG, and TNF-*α*. There was no evidence that dietary treatment effected IgA, IgG, or TNF-*α* concentrations. The effect of sampling day was significant (*P* < 0.05), where circulating IgA and TNF-*α* was increased and IgG was decreased from days 0 to 14. In summary, there is potential for a blend of formic acid and GML to improve growth performance immediately post-weaning without negatively impacting fecal consistency. Formic acid and GML alone or in combination did not impact fecal microbial populations or serum immune parameters.

## INTRODUCTION

The period immediately post-weaning is a crucial time in swine production, as pigs undergo many physiological, dietary, and environmental changes (Zheng et al., 2021). Young piglets who have been switched from a milk diet to solid feed endure damage to the intestinal villi and are unable to properly regulate gastric pH resulting in reduced digestive enzyme secretion and decreased nutrient absorption (Wei et al., 2021). Also during this time, pigs are comingled and potentially exposed to pathogens without a fully-functioning immune system which can lead to post-weaning diarrhea (PWD), one of the most detrimental diseases in the swine industry (Campbell et al., 2013). Collectively, these challenges can severely impact the overall health and growth performance of the animal. One solution to this challenge is the dietary addition of high levels of zinc in the form of zinc oxide (ZnO) during the immediate post-weaning period. When ZnO is included at pharmacological rates near 2,000 to 4,000 ppm, well above the 150 ppm requirement by the pig, a drastic improvement in growth performance and a reduction in PWD have been observed (Hill et al., 2001; Shelton et al., 2011). Despite the effectiveness of pharmacological rates of ZnO, a large portion is excreted in swine manure, which is problematic when the manure is applied to soil as fertilizer because of pollution concerns (Carlson et al., 2004; Jongbloed and Lenis, 1998; Poulsen and Larsen, 1995). Additionally, several studies have linked ZnO supplementation to antimicrobial resistant genes in bacterial pathogens of both animal and human importance, like *E. coli* and *Salmonella* (Ciesinski et al., 2018; Vahjen et al., 2015; Yazdankhah et al., 2014). Due to these concerns, regulatory bodies have begun closely monitoring ZnO application in swine feeding, specifically with the European Union limiting the total Zn concentrations in the diet to 150 ppm beginning in July 2022 (Commission, 2003, 2016). Data suggests that at these levels, the prophylactic effects traditionally seen in the post-weaning period will be diminished; thus, there is a pressing need for ZnO alternatives moving forward (Sales, 2013).

Dietary acidifiers have become a promising solution for preventing PWD and improving growth performance in a world without pharmacological ZnO. While these feed additives were initially used as a preservative, recent work has shown a multitude of positive responses in growth performance, nutrient digestibility, and gut health of weanling pigs (Liu et al., 2018; Tugnoli et al., 2020; Walsh et al., 2007). A variety of organic and inorganic acids and their salts have been studied as alternatives to ZnO or antibiotics, but their precise mechanism of action has yet to be elucidated. Inclusion of formic acid and its derivatives has resulted in improvements in performance, likely due to the reduction in stomach pH and feed buffering capacity, resulting in increased pepsin activation and digestibility of proteins and amino acids (Luise et al., 2020). Additionally, the piglet microbiome is a complex environment of organisms that plays a large role in the animal’s ability to adapt post-weaning. Diet composition represents one of the primary mechanisms to rapidly change the microbial composition along the gastrointestinal (GI) tract. As an example, inclusion of acidifiers like formic acid can limit the colonization of pathogenic bacteria and proliferate commensal microorganisms (Luise et al., 2017). Beyond improving growth performance during the post-weaning period, an emphasis on strengthening the piglet’s immune system to prevent clinical disease is of great importance. Medium-chain fatty acids, those of 6 to 12 carbons in length, have been shown to improve immune system development in young animals and act as viral and bacterial pathogen-mitigants in feed (Jackman et al., 2020). Specifically, the monoglyceride, glycerol monolaurate (GML), has demonstrated the ability to act as a potent immunomodulator in the gut and exhibit antimicrobial properties that could limit the colonization of pathogenic bacteria along the GI tract (Lan et al., 2021; Papadopoulos et al., 2022). Despite numerous studies evaluating acidifiers in the post-weaning period, results are highly variable due to inconsistencies in inclusion rates, age and existing health status of pigs, and response criteria evaluated. Generally, a blend of acids tends to be more effective when considering their use in nursery pig diets. Formic acid and GML are of particular interest since formic acid exerts its primary effects in the stomach, while GML tends to be most beneficial within the small intestine suggesting that the two may act synergistically (Luise et al., 2020; Ramırez et al., 2001). Experiments have been conducted looking at formic acid and GML separately, but there is extremely little data evaluating the two as a blend, despite this combination being available within commercial feed additives. Thus, our objective was to evaluate feeding formic acid and GML alone, or in combination on growth performance, fecal microbiota, fecal consistency, and immune function of weanling pigs. Specifically, this study aimed to determine if these acids could provide additive effects when fed as a blend.

## MATERIALS AND METHODS

### Animals and Diets

All experimental procedures adhered to guidelines for the ethical and humane use of animals for research according to the Guide for the Care and Use of Agricultural Animals in Research and Teaching (FASS, 2012) and were approved by the Institutional Animal Care and Use Committee at Kansas State University (IACUC #4036). The experiment was conducted at the Kansas State University Swine Teaching and Research Center in Manhattan, Kansas.

A total of 350 pigs (DNA 400 × 200; Columbus, NE, initially, 5.67 ± 0.06 kg BW) were weaned at an average of 21 days of age and used in a 42-day experiment. Weaning was considered day 0 of the trial and, at this point, pigs were individually weighed and allotted to pens in a completely randomized design. There were 5 pigs per pen and 14 replicate pens per treatment. Each pen had tri-bar floors (1.5 × 1.5 m) and was equipped with a 4-hole dry self-feeder and nipple waterer to supply *ad libitum* access to feed and water. At weaning, pigs were randomized to pens, and pens randomly assigned to dietary treatments. Diets were formulated and manufactured in three dietary phases (phase 1 = days 0 to 7; phase 2 = days 8 to 28; phase 3 = days 29 to 42) such that experimental diets were fed from days 0 to 28, and a common diet was fed to all pigs from days 29 to 42. Diets were formulated to meet or exceed (NRC, 2012) requirements (Table 1) and contained no antimicrobials. Pigs treated with antimicrobials for any reason were immediately removed from the experiment. Treatments were as follows: 1) negative control (**CON;** standard nursery diet containing 150 ppm Zn from trace mineral premix and no acidifier); 2) control diet with 3,000 ppm added zinc from ZnO included in phase 1 and 2,000 ppm added zinc from ZnO included in phase 2 **(ZnO)**; 3) control diet with 0.7% formic acid (**FA;** Amasil® NA; BASF, Florham, NJ); 4) control diet with 0.18% glycerol monolaurate (**GML;** Natural Biologics GML, Natural Biologics, Newfield, NY); and 5) control diet with a 1.0% blend of formic acid, sodium diformate, and glycerol monolaurate (**FORMI;** FORMI 3G, ADDCON GmbH, Bitterfeld-Wolfen, Germany). All test ingredients were included at the expense of corn in the diet. Individual pig weights and feed disappearance were measured on days 0, 7, 14, 21, and 28, with pen weights collected on days 35 and 42 to calculate average daily gain (ADG), average daily feed intake (ADFI), and feed efficiency (G:F).

**Table 1.** Composition of phase 1, phase 2, and phase 3 basal diets (as-fed basis)

Item	Dietary phase^1^		
	Phase 1	Phase 2	Phase 3
Ground corn	40.08	45.98	65.50
Soybean meal, 47.5% CP	17.30	22.75	28.45
Corn DDGS, 7.5% oil	5.00	10.00	—
Spray dried whey	25.00	10.00	—
Fish meal	3.00	—	—
Enzymatically treated soybean meal^2^	—	4.5	—
Spray dried bovine plasma	4.00	—	—
Fat	3.00	3.00	2.00
Calcium carbonate, 38.5% Ca	0.65	0.85	0.75
Monocalcium phosphate, 21.5% P	0.55	1.00	1.10
Salt	0.30	0.55	0.60
L-Lys HCL	0.35	0.50	0.55
DL-Met	0.15	0.17	0.21
L-Thr	0.13	0.18	0.23
L-Trp	0.03	0.04	0.05
L-Val	0.07	0.08	0.16
Zinc oxide	Varied	Varied	—
Formic acid^3^	Varied	Varied	—
Glycerol monolaurate (GML), 90% purity^4^	Varied	Varied	—
Formic acid and GML, 1.0% blend^5^	Varied	Varied	—
Vitamin premix w/ phytase^6^	0.25	0.25	0.25
Trace mineral premix^7^	0.15	0.15	0.15
Total	100.00	100.00	100.00
Calculated analysis^8^			
SID amino acids, %			
Lys	1.40	1.35	1.30
Ile:Lys	57	57	53
Leu:Lys	110	118	111
Met:Lys	36	35	36
Met and Cys:Lys	57	57	57
Thr:Lys	63	64	63
Trp:Lys	19.3	20	19.3
Val:Lys	70	70	70
His:Lys	33	34	35
Total Lys, %	1.54	1.49	1.43
ME, kcal/kg	3,440	3,353	3,383
NE, kcal/kg	2,594	2,449	2,534
SID Lys:NE, g/Mcal	5.39	5.51	5.13
CP, %	21.1	21.2	19.9
Ca, %	0.74	0.75	0.65
P, %	0.69	0.67	0.61
STTD P, %	0.48	0.42	0.49

^1^Treatment diets were fed to 350 pigs [DNA 400 × 200 (Columbus, NE); initially 5.67 ± 0.06 kg BW] for 28 days in a 2-phase feeding program with 5 pigs per pen and 14 pens per treatment. A common phase 3 diet was fed to all pigs from days 29 to 42.

^2^HP300 (Hamlet Protein, Findlay, OH).

^3^Formic acid included at 0.70% in phases 1 and 2 (Amasil-NA; BASF Corp., Florham, NJ).

^4^Glycerol monolaurate (guaranteed ≥ 95% purity) included at 0.18% in phase 2 and 2 (GML; Natural Biologics, Newfield, NY).

^5^A 1.0% blend of formic acid and glycerol monolaurate was included in phases 1 and 2 (FORMI-3G; ADDCON GmbH, Bitterfeld-Wolfen, Germany).

^6^Premix provided per kg of premix: 4,409,249 IU vitamin A; 551,156 IU vitamin D3; 17,637 IU vitamin E; 1,764 mg vitamin K; 15.4 mg vitamin B12; 19,842 mg niacin; 11,023 mg pantothenic acid; and 3,307 mg riboflavin.

^7^Premix provided per kg of premix: estimated release of 0.12% STTD P; 110 g Fe from iron sulphate; 110 g Zn from zinc sulfate; 26.4 g Mn from manganese oxide; 11 g Cu from copper sulphate; 198 mg I from calcium iodate; 198 mg Se from sodium selenite.

^8^ NRC (2012).

### Chemical Analysis

Complete diet samples were collected from 10 different feeders per dietary treatment on days 0 and 21 and composite subsamples were analyzed for nutrient composition (Table 2). Assays included DM (method 930.15; AOAC, 2007), crude protein (CP) as N × 6.25 using the combustion method (Nitrogen Determinator; model TruMac N, Leco Corporation, St. Joseph, MI; method 990.03; AOAC, 2007), acid detergent fiber (ADF) (ANKOM Tech. Method 200), Ca (method 985.01; AOAC, 2006), P (method 985.01; AOAC, 2006), Zn (method 985.01; AOAC, 2006), NE, gain (by calculation), ME (by calculation), and fatty acid profile (method 996.06; AOAC, 2010). Diet analyses are presented in Table 3.

**Table 2.** Analyzed composition of phase 1 and phase 2 experimental diets (as-fed basis)^1^

Analyzed composition, %^3^	Dietary treatment^2^				
	CON	ZnO	FA	GML	FORMI
Phase 1					
DM, %	88.04	87.89	88.11	88.31	87.95
CP, %	20.30	20.70	21.40	21.00	21.00
Fat, %	5.14	5.41	5.48	5.51	5.50
ADF, %	2.50	2.40	2.90	2.50	2.50
NE gain, Mcal/kg	0.56	0.55	0.55	0.55	0.55
ME, Mcal/kg	1.38	1.37	1.37	1.39	1.37
Calcium, %	0.75	0.80	0.83	0.86	0.81
Phosphorous, %	0.67	0.70	0.71	0.73	0.73
Zinc, ppm	168	2,840	309	113	139
Lauric acid (C:12), g/100 g	<0.01	<0.01	<0.01	0.10	0.13
Phase 2					
DM, %	86.92	87.11	86.65	87.04	86.79
CP, %	18.50	19.80	19.60	18.50	20.20
Fat, %	4.74	5.48	4.88	4.98	4.63
ADF, %	3.70	3.9	4.00	3.80	3.80
NE gain, Mcal/kg	0.54	0.54	0.54	0.55	0.53
ME, Mcal/kg	1.36	1.36	1.35	1.38	1.34
Calcium, %	0.85	0.78	0.66	0.58	0.64
Phosphorous, %	0.63	0.7	0.63	0.53	0.65
Zinc, ppm	284	1,900	188	146	192
Lauric acid (C:12), g/100 g	<0.01	<0.01	<0.01	0.13	0.17

^1^Treatment diets were fed to 350 pigs [DNA 400 × 200 (Columbus, NE); initially 5.67 ± 0.06 kg BW] for 28 days in a 2-phase feeding program with 5 pigs per pen and 14 pens per treatment.

^2^Dietary treatments included: basal diet (CON); basal diet with 3,000 ppm added Zn from ZnO in phase 1 and 2,000 ppm added Zn from ZnO in phase 2; basal diet with 0.70% formic acid (Amasil-NA, BASF Corp., Florham, NJ); basal diet with 0.18% glycerol monolaurate (GML guaranteed ≥ 95% purity; Natural biologics, Newfield, NY); basal diet with 1.0% blend of formic acid and GML (FORMI-3G, ADDCON GmbH, Bitterfeld-Wolfen, Germany).

^3^Complete diet samples were collected from the same 10 randomly selected feeders on days 0 and 21. Samples were pooled by day and subsampled, then submitted to Midwest Laboratories (Omaha, NE) for proximate and fatty acid analysis.

**Table 3.** Analyzed composition of phase 3 common diet (as-fed basis)^1^

Analyzed composition, %^2^	Diet
DM, %	87.23
CP, %	19.50
Fat, %	4.53
ADF, %	3.20
NE gain, Mcal/kg	0.55
ME, Mcal/kg	1.38
Calcium, %	0.61
Phosphorous, %	0.57
Zinc, ppm	136.0
Lauric acid (C:12), g/100 g	<0.01

^1^A common diet was fed to 350 pigs [DNA 400 × 200 (Columbus, NE); initially 5.67 ± 0.06 kg BW] from days 29 to 42 with 5 pigs per pen and 14 pens per treatment.

^2^Complete diet samples were collected from the same 10 randomly selected feeders on days 28 and 42. Samples were pooled and subsampled, then submitted to Midwest Laboratories (Omaha, NE) for proximate and fatty acid analysis.

### Fecal Dry Matter and Microbiota Analysis

Fecal samples were collected from the same 3 randomly selected pigs per pen on days 7, 14, 21, and 28 for analysis of fecal DM. Briefly, a sterile, cotton-tipped applicator was gently inserted into the rectum to stimulate defecation. Samples were pooled on a per pen basis to form one composite sample for each collection point and stored at −20 °C until analysis. To determine fecal DM, samples were dried for 48 h at 55 °C in a forced air oven.

On days 0 and 14, fresh fecal samples were collected from an additional one pig per pen (*n* = 14 pigs/treatment, 70 pigs total) for fecal microbiota analysis. The same pig was sampled at each time point by inserting a sterile, cotton-tipped applicator into the rectum to stimulate defecation. Samples were collected into sterile, DNA-free centrifuge tubes and stored at −80 °C until shipment to a commercial laboratory (MR DNA, Shallowater, TX) for DNA extraction and taxonomic analysis.

Genomic DNA was isolated from each sample using the PowerSoil DNA Isolation Kit (Qiagen Inc., Valencia, CA). Approximately 250 mg of fecal matter was added to a PowerBeads (Qiagen Inc.) tube and homogenized using the PowerLyzer (Qiagen Inc.) to induce cell lysis. The purified DNA was then eluted and stored at −20 °C until PCR amplification. The 16S universal primers 515F (GTGYCAGCMGCCGCGGTAA) and 806R (GGACTACNVGGGTWTCTAAT) were utilized to amplify 16S gene of samples on the Illumina NovaSeq (Illumina Inc., San Diego, CA) via the bTEFAP DNA analysis service originally described by (Dowd et al., 2008a). Each sample underwent a single-step 35 cycle PCR using the HotStarTaq Plus Master Mix Kit (Qiagen) under the following conditions: 95 °C for 5 min, followed by 30 cycles of 95 °C for 30 s; 53 °C for 40 s and 72 °C for 1 min; after which a final elongation step at 72 °C for 10 min was performed. Following PCR, all amplicon products from different samples were mixed in equal concentrations and purified using calibrated solid phase reversible immobilization methodology (SPRI) beads. Samples were sequenced utilizing the Illumina NovaSeq (Illuminia Inc., San Diego, CA) chemistry following manufacturer’s protocols.

Data processing was conducted using a proprietary analysis pipeline (MR DNA, Shallowater, TX). Sequences were depleted of primers, short sequences (<150 bp) were removed, and sequences with ambiguous base calls removed. Sequences were quality filtered using a maximum expected error threshold of 1.0 and dereplicated. The dereplicated or unique sequences were denoised, and unique sequences identified with sequencing or PCR point errors were removed, followed by chimera removal, thereby providing a denoised sequence or operational taxonomic unit (**OTU**). Final OTUs were taxonomically classified using BLASTn (Altschul et al., 1990) against a curated database derived from NCBI (www.ncbi.nlm.nih.gov). Alpha and beta diversity analyses were conducted as previously described by (Dowd et al., 2008b; Edgar, 2010) using Qiime v.2 (Bolyen et al., 2018). After stringent quality sequence curation, a total of 4,037,671 sequences identified within the Bacteria and Archaea domains were utilized for final microbiota analyses. The average reads per sample was 29,258. For alpha and beta diversity analysis, samples were rarefied to 10,000 sequences.

### Serum Immunoglobulin and Pro-Inflammatory Cytokine Analysis

On days 0 and 14, whole blood samples were collected from one pig per pen (*n* = 14 pigs/treatment, 70 pigs total). The same pig was sampled at each time point. Samples were collected from the jugular vein into sterile 5 mL vacuum-sealed tubes (Monoject Blood Collection Tube, Mansfield, MA) using a 22 *g* × 1ʹʹ blood collection needle (Fisher Scientific, Hampton, NH). Samples were immediately placed on ice until transport to the laboratory for serum separation. Samples were allowed to clot at room temperature for 2 h before being centrifuged at 2,000 × *g* for 10 min at 4 °C. Serum was then recovered from samples, placed into aliquots for each assay, and stored at −80 °C until analysis. Serum was diluted at 1:10,000 and 1:500,000 for analysis of IgA and IgG, respectively. Samples were not diluted for analysis of TNF-α. The intra-assay CV was 6.5%, 3.3%, and 4.1% for IgA, IgG, and TNF-α, respectively. The interassay CV was 8.1%, 5.1%, and 5.6% for IgA, IgG, and TNF-α, respectively. Using an enzyme-linked immunoassay (ELISA), concentrations of tumor necrosis factor-α (TNF-α; R&D Systems, Inc., Minneappolis, MN), immunoglobulin A (IgA; Bethyl Laboratories, Montgomery, TX), and immunoglobulin G (IgG; Bethyl Laboratories) were measured according to the kit manufacturer’s instructions.

### Statistical Analysis

Growth and fecal consistency data were analyzed as a completely randomized design with pen of pigs as the experimental unit. Fecal dry matter data were analyzed as repeated measures over time. All comparisons included Tukey-Kramer multiple comparison adjustments. Additionally, preplanned contrasts were included to compare the means of the negative control and diets containing an additive, as well as the ZnO and acidifier diets. Serum immunoglobulin and pro-inflammatory cytokine data were log transformed before analysis. Data were then analyzed as a completely randomized design with individual pig as the experimental unit. The model included the main effects of dietary treatment, sampling day, and the treatment × day interaction. Data were analyzed using the GLIMMIX procedure of SAS version 9.4 (SAS Institute, Cary, NC).

Fecal microbiota data were analyzed with individual pig as the experimental unit to represent one sample selected from each pen on each sampling day (days 0 and 14). The relative abundance was determined as the total proportion of reads for a sample classified into the specific microbial phyla or family. Samples with a low relative abundance (<0.01%) were excluded from the analysis. Once the relative abundance for each sample was calculated, data were analyzed as a completely randomized design with the model including dietary treatment, sampling day, and their interaction as fixed effects. Data were analyzed as repeated measures given the two sampling time points on days 0 and 14. Statistical comparisons of observed features and Shannon Diversity indices for each treatment group were conducted using Kruskal-Wallis pairwise comparisons. Beta diversity was analyzed using a weighted UniFrac distance matrix with pairwise analysis of similarities (ANOSIM) used to evaluate differences in microbial communities between groups over time. Data were considered significant if *P* < 0.05 and marginally significant if 0.05 < *P* < 0.10.

## RESULTS AND DISCUSSION

### Growth Performance and Fecal Consistency

Growth performance data are presented in Table 4. During the first week post-weaning (days 0 to 7), pigs fed a diet with either ZnO or FORMI had increased (*P* < 0.01) ADG compared to pigs fed the control diet. There was no evidence of differences in ADFI (*P* > 0.05), but pigs fed a diet supplemented with GML had improved feed efficiency (*P* < 0.01) compared to their counterparts fed both the control and FA diets, while pigs fed ZnO or FORMI were intermediate. Similarly, others have reported improvements in feed conversion post-weaning with the addition of MCFA, like GML (Cera et al., 1989; Gebhardt et al., 2020; Hanczakowska et al., 2010). Due to their higher water solubility compared to long chain fatty acids (LCFA), MCFA are hydrolyzed rapidly and easily absorbed in the small intestine (Jackman et al., 2020). This makes them a readily available energy source for the young pig during a time when reduced feed intake can cause energy deficiencies, thus potentially explaining the enhanced feed efficiency seen in the first week of the current experiment. However, this response diminished by dietary phase 2 (days 8 to 28), where we saw a reduction in ADG in pigs fed GML, which appeared to be driven by decreased feed conversion (*P* < 0.0001). While others have reported no effect of GML supplementation on growth performance in the nursery period (Cui et al., 2020; Thomas et al., 2020), the negative growth response that was seen in the current work from d 7 and beyond was unexpected. It is important to note that the addition of some MCFA can pose palatability concerns and have been associated with reductions in feed intake due to their pungent odor (Hanczakowska, 2017). However, the lack of feed intake response in our study does not corroborate this.

**Table 4.** Effect of formic acid and glycerol monolaurate alone or in combination nursery pig growth performance^1^

Item	CON	ZnO^2^	FA^3^	GML^4^	FORMI^5^	SEM	Treatment	Control vs. additives^6^	Zinc oxide vs. acids^7^
BW, kg									
Day 0	5.62	5.69	5.77	5.66	5.61	0.06	0.340	0.335	0.916
Day 7	5.96	6.21	6.09	6.18	6.21	0.06	0.033	0.005	0.501
Day 14	7.30^ab^	7.74^a^	7.29^b^	7.21^b^	7.64^ab^	0.11	0.003	0.171	0.007
Day 21	10.17^bc^	11.40^a^	10.15^bc^	9.54^c^	10.72^ab^	0.18	<0.0001	0.154	<0.0001
Day 28	13.95^bc^	15.34^a^	13.97^bc^	13.18^c^	14.58^ab^	0.21	<0.0001	0.166	<0.0001
Day 35	18.69^b^	20.16^a^	18.59^bc^	17.83^c^	19.15^b^	0.22	<0.0001	0.328	<0.0001
Day 42	23.77^b^	25.14^a^	23.24^bc^	22.50^c^	24.24^ab^	0.32	<0.0001	0.975	<0.0001
Phase 1 (days 0 to 7)									
ADG, kg/d	0.05^b^	0.09^a^	0.06^ab^	0.07^ab^	0.09^a^	0.02	0.003	0.003	0.146
ADFI, kg/d	0.11	0.13	0.13	0.11	0.14	0.01	0.162	0.146	0.619
G:F	0.40^b^	0.62^ab^	0.45^b^	0.69^a^	0.62^ab^	0.06	0.002	0.003	0.606
Phase 2 (days 8 to 28)									
ADG, kg/d	0.38^b^	0.42^a^	0.38^ab^	0.33^c^	0.40^ab^	0.01	< 0.0001	0.642	<0.0001
ADFI, kg/d	0.58	0.63	0.58	0.57	0.59	0.02	0.058	0.344	0.007
G:F	0.65^a^	0.67^a^	0.64^a^	0.59^b^	0.68^a^	0.01	<0.0001	0.446	0.026
Overall treatment (days 0 to 28)									
ADG, kg/d	0.29^bc^	0.33^a^	0.30^bc^	0.27^c^	0.32^ab^	0.01	<0.0001	0.247	<0.0001
ADFI, kg/d	0.46	0.50	0.47	0.46	0.48	0.01	0.120	0.291	0.027
G:F	0.64^a^	0.67^a^	0.63^ab^	0.59^b^	0.67^a^	0.01	<0.0001	0.947	0.008
Common phase 3 (days 29 to 42)									
ADG, kg/d	0.70	0.70	0.68	0.67	0.69	0.02	0.247	0.212	0.137
ADFI, kg/d	0.98	0.99	0.98	0.94	0.98	0.03	0.555	0.938	0.400
G:F	0.73	0.71	0.69	0.72	0.71	0.02	0.732	0.325	0.921
Overall experiment (days 0 to 42)									
ADG, kg/d	0.43^ab^	0.45^a^	0.42^bc^	0.40^c^	0.44^ab^	0.01	<0.0001	0.929	0.001
ADFI, kg/d	0.63	0.66	0.64	0.62	0.64	0.04	0.846	0.879	0.860
G:F	0.68	0.69	0.66	0.65	0.69	0.01	0.139	0.896	0.017

^1^A total of 360 weanling pigs (DNA 200 × 400, initially 5.67 ± 0.06 kg BW) were used in a 42-d growth study with 5 pigs/pen and 14 replicates/treatment.

^2^Added Zn in the form of zinc oxide was provided at 3,000 ppm in phase 1 and at 2,000 ppm in phase 2.

^3^Formic acid (Amasil-NA; BASF Corp. Florham, NJ) was included in the diet at 0.70% in both phase 1 and phase 2.

^4^Glycerol monolaurate (guaranteed ≥ 95% purity) (Natural Biologics, Newfield, NY) was included in the diet at 0.18% in both phase 1 and phase 2.

^5^FORMI-3G (Addcon Gmbh, Bitterfeld-Wolfen, Germany) was included in the diet at 1.0% in both phase 1 and phase 2.

^6^Contrast statement used to evaluate means from pigs fed the Control compared to those fed ZnO, Formic Acid, Glycerol Monolaurate, and FORMI-3G.

^7^Contrast statement used to evaluate means from pigs fed ZnO compared to those fed Formic Acid, Glycerol Monolaurate, and FORMI-3G.

^abc^Means within a row that do not share a common superscript differ (*P* < 0.05).

From days 8 to 28, pigs fed ZnO had increased ADG compared to control-fed pigs (*P* = 0.01), while those fed FA or FORMI were intermediate. Despite a marginally significant treatment effect for feed intake (*P* = 0.06), when a contrast statement was used to directly compare ZnO to the three acidifiers, we saw a significant feed intake response (*P* < 0.01), where pigs fed ZnO had increased ADFI. During the entirety of the treatment period (days 0 to 28) pigs fed ZnO had increased ADG compared to those fed the control, FA, and GML diets, while pigs fed FORMI were intermediate (*P* < 0.0001). This coincides with previous literature, where the growth enhancements from feeding added ZnO are well documented (Hill et al., 2001; Sales, 2013). Feed efficiency was negatively impacted by feeding GML (*P* < 0.0001) compared to a control, ZnO, or FORMI, while feeding FA alone yielded an intermediate response. A common phase 3 diet was fed to all pigs from days 29 to 42, where no evidence of differences were observed for ADG, ADFI, or G:F (*P* ≥ 0.25). Overall (days 0 to 42), a reduction in ADG was seen in pigs fed GML compared to a control, ZnO, or FORMI, while those fed FA were intermediate (*P* < 0.0001). No evidence of differences in ADFI or G:F were observed for the overall experiment (*P* ≥ 0.14).

Despite many studies evaluating formic acid and GML alone, experiments investigating their efficacy together are scarce. The use of formic acid and its salts to improve growth performance in the post-weaning period was recently reviewed by Luise et al. (2020). While numerous studies have shown favorable growth responses to formic acid, the precise mode of action is still not clear. Literature suggests several mechanisms by which formic acid can improve weanling pig growth performance including the reduction of gastric pH, increased nutrient digestibility, and alterations in the intestinal microbiome leading to increased SCFA production and energy metabolism by the pig (Partanen and Mroz, 1999). During the entirety of this experiment, we saw no evidence that formic acid inclusion impacted piglet growth compared to a negative control. Parameters like gut pH and nutrient digestibility were not measured in our study to explain the lack of response. When formic acid was fed together with GML, there appeared to be no impact on piglet growth compared to a negative control. Within the literature, responses seen when formic acid or MCFA have been included are extremely variable and highly dependent on other factors such as inclusion rate, other diet components, piglet age, and health status. In our experimental diets containing solely formic acid or GML, the concentrations of each acid used were calculated to equal the levels of both formic acid and GML present within the 1% blend (FORMI), in effort to detect any additive effects if present. In the first week post-weaning, there was no evidence of differences in growth performance between pigs fed formic acid and GML alone or a blend of the two. However, by week 2, pigs fed FORMI had improved ADG compared to those fed GML alone, and this response was consistent throughout the remainder of the trial, which may indicate that the effect of GML can be enhanced when paired with formic acid.

**Figure 1.**
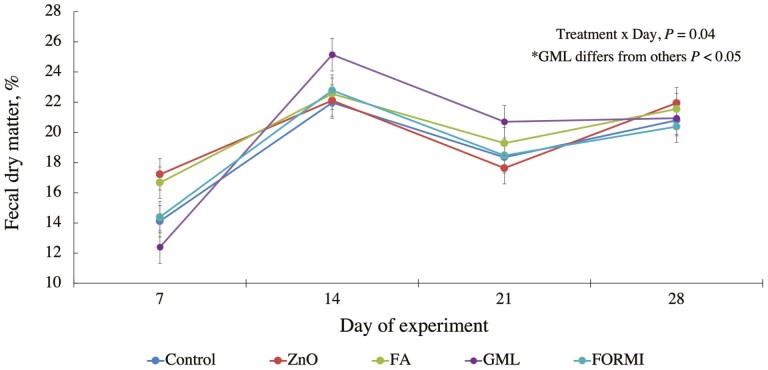
Effect of dietary treatments on weanling pig fecal dry matter percentage. On days 7, 14, 21, and 28 a fresh fecal sample was collected from the same 3 pigs per pen. Samples were pooled and dried for 48 h in a 55 °C forced air oven. Dietary treatments included basal diet (CON); basal diet with 3,000 ppm added Zn from ZnO in phase 1 and 2,000 ppm added Zn from ZnO in phase 2 (ZnO); basal diet with 0.70% formic acid (FA; Amasil-NA, BASF Corp., Florham, NJ); basal diet with 0.18% glycerol monolaurate (GML; guaranteed ≥ 95% purity; Natural biologics, Newfield, NY); basal diet with 1.0% blend of formic acid and GML (FORMI; FORMI-3G, ADDCON GmbH, Bitterfeld-Wolfen, Germany).

Fecal dry matter data are presented in Figure 1. A treatment × day interaction was observed for fecal DM (*P* = 0.04). On day 7, pigs fed a diet with GML had a lower fecal DM % (*P* = 0.04) compared to pigs fed all other treatments. Interestingly, by day 14, this response had shifted, and pigs fed GML had a higher fecal DM % compared to their counterparts, indicating firmer fecal consistency. This response was observed through day 21, but by day 28 there were no differences in fecal DM. Feeding pharmacological levels of ZnO has been shown to reduce post-weaning diarrhea because of its ability to limit the colonization of pathogenic bacteria along the GI tract (Hu et al., 2013a, 2013b). Certain MCFA, like lauric acid (C12:0) are known to have antimicrobial properties, and the efficacy is considerably greater when in monoglyceride form (Bergsson et al., 2002; Kabara et al., 1972). Meanwhile, Pettigrew (2006) reported that due to the reduction in gastric pH from formic acid supplementation, alterations in the bacterial population within the stomach and subsequent portions of the GI tract could be achieved. However, literature is highly variable as it relates to the efficacy of acidifiers to control PWD, primarily due to differences in inclusion rates and the blends of acids used. Within the first week post-weaning, pigs fed GML had a reduced fecal DM percentage. It is difficult to clearly explain this response; however, we speculate that a change in intestinal bacterial populations not observed in fecal analysis, or an incorrect electrolyte balance could be contributing factors. However, while we observed statistically significant differences in fecal DM percentage across treatments, the numeric difference was still quite small, making it challenging to draw conclusions about the incidence of clinical diarrhea. Further investigation into the mode of action of dietary acidifiers is needed to fully elucidate their impacts on piglet fecal consistency.

### Fecal Microbiota

**Figure 2.**
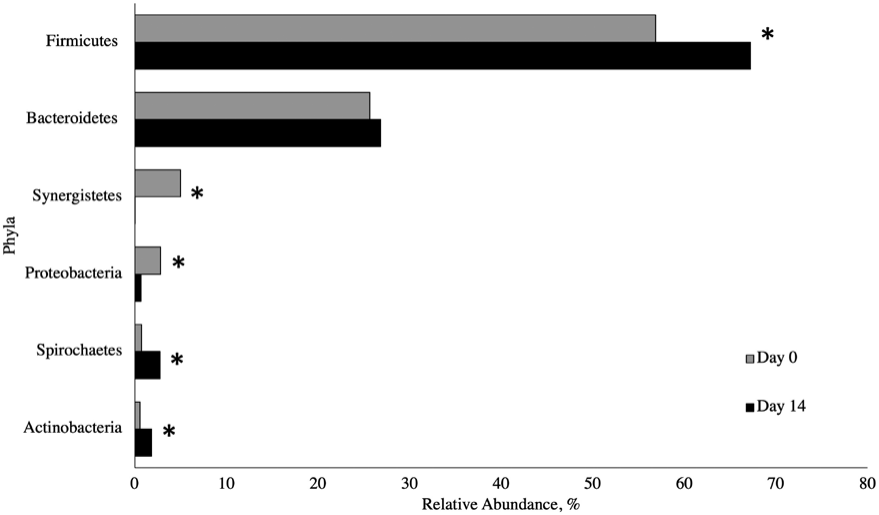
Relative abundance of microbial phyla by day presented as a proportion of all reads for a specific sample classified into the designated phyla. On days 0 and 14, a fresh fecal sample was collected from the same 70 randomly selected pigs (1 pig per pen, 14 pens per treatment) for analysis of fecal microbiota. Samples were stored at −80 °C until DNA extraction and taxonomic analysis. Samples were analyzed using 16s rDNA sequencing. A ≥0.01% relative abundance threshold was set and samples not meeting this criterion were omitted from analysis. Dietary treatment did not significantly impact (*P* > 0.05) fecal microbial populations at the phyla level. A sampling day effect (*P* ≤ 0.01) was observed, where the mean relative abundance of Actinobacteria, Spirochaetes, and Firmicutes significantly increased from days 0 to 14, while the relative abundance of Proteobacteria and Synergistetes significantly decreased. ^*^Main effect of day, *P* < 0.05. The relative abundance of Bacteroidetes did not differ significantly from days 0 to 14 (*P* = 0.71).

Most sequences were classified into the phyla Firmicutes and Bacteroidetes on days 0 and 14 (Figure 2), which is consistent with previous literature studying microbial populations at different portions of the pig gastrointestinal tract (Gebhardt et al., 2020; Li et al., 2018; Yang et al., 2017). We observed no evidence of differences (*P* > 0.05) in microbial phyla as a result of dietary treatment; however, a significant sampling day effect (*P* ≤ 0.01) was seen for several of the analyzed phyla. The mean relative abundance of Actinobacteria, Spirochaetes, and Firmicutes increased from days 0 to 14, while the relative abundance of Proteobacteria and Synergistetes decreased. The relative abundance of Bacteroidetes did not differ from days 0 to 14 (*P* = 0.71). Our data do not suggest that feeding formic acid and GML altered the microbial composition at the phya level, rather a change over time was observed. This alteration in the microbes present immediately after weaning until 2 weeks post-weaning was expected, as it is well understood that the microbiome shifts drastically with time and along the GI tract (Isaacson and Kim, 2012). In contrast to the current work, Yang et al. (2021) reported an increase in the relative abundance of Bacteroidetes over time. These authors also found that microbes in the phyla Proteobacteria were highly abundant after birth, but decreased post-weaning, which coincides with our data.

**Figure 3.**
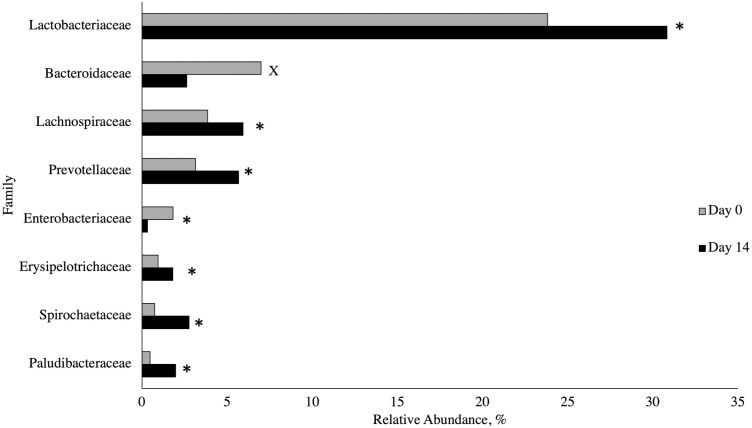
Relative abundance of microbial families by day presented as a proportion of all reads for a specific sample classified into the designated family. On days 0 and 14, a fresh fecal sample was collected from the same 70 randomly selected pigs (1 pig per pen, 14 pens per treatment) for analysis of fecal microbiota. Samples were stored at −80 °C until DNA extraction and taxonomic analysis. Samples were analyzed using 16s rDNA sequencing. A ≥0.01% relative abundance threshold was set and samples not meeting this criterion were omitted from analysis. A sampling day effect (*P* ≤ 0.01) was observed at the family level, where a significant increase (*P* ≤ 0.01) in the families *Paludibacteraceae*, *Spirochaetaceae*, *Erysipelotrichaceae*, *Prevotellaceae*, *Lachnospiraceae*, and *Lactobacteriaceae* was observed from days 0 to 14, while the family *Enterobacteriaceae* significantly decreased (*P* = 0.03). ^*^Main effect of day, *P* < 0.05. The family *Bacteroidaceae* tended to decrease (*P* = 0.08) from days 0 to 14. ^x^Main effect of day, 0.05 < *P* ≤ 0.10.

Microbial populations at the family level are shown in Figure 3. A marginally significant treatment × day interaction (*P* = 0.07) was observed for the family *Ruminococcaceae*, where pigs fed the CON and ZnO diets had a decreased relative abundance from days 0 to 14, while those fed FA, GML, or FORMI had increased *Ruminococcaceae*. A dietary treatment effect was seen for the relative abundance of *Clostridiaceae* (*P* = 0.01), where samples from pigs fed ZnO had increased *Clostridiaceae* compared to those fed CON or FA, with pigs fed a diet with GML or FORMI being intermediate. When considering the piglet microbiota, the weaning transition is associated with dysbiosis that can be identified by reduced abundance of microbes within the Clostridia class, like *Clostridiaceae* (Gresse et al., 2017). Additionally, in dogs, *Clostridiaceae* are associated with increased protein and fat digestibility (Bermingham et al., 2017); however, nutrient digestibility was not measured in the current work. Sampling day impacted the relative abundance of several families. An increase (*P* ≤ 0.01) in the families *Paludibacteraceae, Spirochaetaceae, Erysipelotrichaceae, Prevotellaceae, Lachnospiraceae,* and *Lactobacteriaceae* was observed from days 0 to 14, while the family *Enterobacteriaceae* decreased (*P* = 0.03) and *Bacteroidaceae* tended to decrease (*P* = 0.08). This is consistent with Frese et al. (2015), who studied changes in the microbiome from suckling to post-weaning.

**Figure 4.**
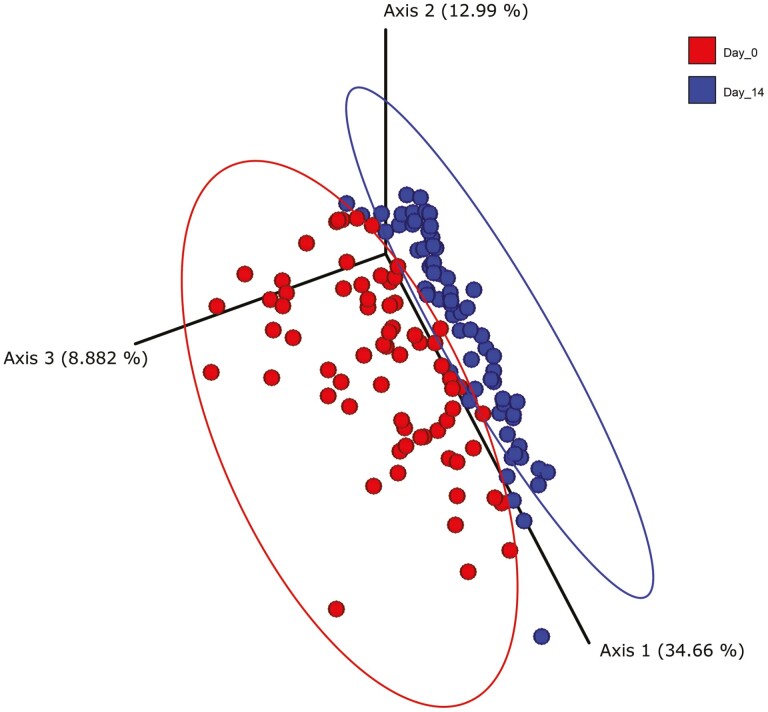
Principal coordinate plot of the microbial community structure between samples over time. On days 0 and 14, a fresh fecal sample was collected from the same 70 randomly selected pigs (1 pig per pen, 14 pens per treatment) for analysis of fecal microbiota. Samples were stored at −80 °C until DNA extraction and taxonomic analysis. Samples were analyzed using 16s rDNA sequencing. A ≥ 0.01% relative abundance threshold was set and samples not meeting this criterion were omitted from analysis. The microbial community structure, or Beta diversity, was analyzed using a weighted UniFrac distance matrix. A principal coordinate plot was used to visualize the data in a distance matrix, and pairwise analysis of similarities (ANISOM) was utilized to determine significant differences between microbial communities over time. Samples collected on day 0 have a clear phylogenetic assemblage that differs significantly from samples collected on day 14 (*P* < 0.01). Primary vector explains 34.6% of the variation between groups. The first 3 vectors together exhibit 56.5% of the variation among the groups.

The alpha diversity of microbes present within a sample are commonly assimilated according to the richness (quantity) and evenness (dispersion) (Prehn-Kristensen et al., 2018). Based on the number of observed features, the alpha diversity of the samples from pigs fed CON, FA, GML, or FORMI was less (*P* < 0.05) on day 0 compared to day 14, while there was no evidence of a difference in the alpha diversity from pigs within the ZnO treatment. Generally, a higher diversity is indicative of a more healthy microbiome (Lozupone et al., 2012). Our data indicate that supplementing pharmacological ZnO had the least impact on the number of microbes present in fecal samples. Conversely, Vahjen et al. (2011) reported an increase in these parameters when weanling pigs were supplemented with 3,000 ppm ZnO, but these samples were taken from the ileum rather than fecal samples as seen in the current work. However, it is important to not only consider the quantity of microbes, but also the dispersion of them according to taxonomic classification. The analysis of the Shannon Diversity indices, which accounts for species evenness in addition to species richness, only detected a significant difference within samples from pigs fed either GML or FORMI over time (*P* < 0.05), which suggests that GML supplemented alone or with formic acid can alter the structure of the microbial community in piglet feces. Beyond evaluating differences in microbial populations within a given sample, comparison between samples is considered Beta diversity (Liu et al., 2021). No evidence of differences in beta diversity was seen across dietary treatments (*P* ≥ 0.25). However, there was a difference (*P* < 0.01; Figure 4) in the beta diversity of all samples between days 0 and 14, which further validates the taxonomic differences over time within our samples.

It is important to consider that it is nearly impossible to determine a ‘standard’ microbiota composition post-weaning, given the literature is extremely inconsistent due to differences in genetics, diet composition, weaning age, environment, and experimental methodologies. The microbiota differs substantially throughout the gastrointestinal tract (Zhao et al., 2015), therefore it is difficult to elucidate how dietary acidifiers like formic acid or GML impact the microbiome as a whole. Fecal analysis may only represent a small portion of the microbial population present within an animal, so there could be drastic differences in these samples compared to those from other areas such as the intestine or stomach. We would expect both formic acid and glycerol monolaurate to be most effective in the upper GI tract, therefore, only analyzing fecal microbes is certainly a limitation of the current work. Future studies should focus on how these additives can alter the intestinal microbiota specifically. The lack of treatment differences in our data suggest that the changes in growth performance may not have been directly caused by alterations in the piglet fecal microbial populations.

### Serum IgA, IgG, and TNF-α Concentrations

There was no evidence of a dietary treatment × sampling day interaction or a main effect of dietary treatment for any serum parameters (*P* > 0.05), therefore these data are not shown. However, sampling day impacted IgA, IgG, and TNF-α concentrations (*P* < 0.05), therefore these data are presented as the calculated change in the targeted parameter from days 0 to 14. We observed an increase (*P* < 0.05) in serum IgA and TNF-α levels from days 0 to 14 for all pigs, while the concentration of IgG decreased (*P* < 0.05), regardless of dietary treatment.

The weaning transition predisposes piglets to immunological challenges and intersects with the time at which passive immunity from the sow’s milk declines (de Groot et al., 2021). Therefore, strengthening the pigs immune system post-weaning is crucial to maintaining health and growth performance. Others have shown GML to exhibit strong immunomodulatory properties by decreasing the production of certain cytokines and reducing gut inflammation (Zhang et al., 2016, 2018). While formic acid is not known to directly impact immune function, the symbiotic relationship between the gut microbiota and mucosal immunity is important to consider. Formic acid may indirectly enhance the weaned piglets immune response by altering the GI microbial populations to a more favorable environment (Luise et al., 2020). While we did not observe a significant effect of dietary treatment on serum immune parameters, other studies have reported different findings. Ren et al. (2020) challenged 25-day old piglets with enterotoxigenic *E. coli* and found that a blend of formic acid and monolaurin resulted in lower plasma TNF-α by downregulating the expression of toll-like receptor 4 (TLR4), ultimately attenuating gut inflammation triggered by the ETEC. Inclusion levels of formic acid and GML were 0.60% and 0.20%, respectively, while the current study included formic acid at 0.70% and GML at 0.18%. With similar levels of both formic acid and GML, we did not observe a significant change in serum immunity; however, Ren et al. (2020) collected samples post ETEC challenge and pigs in the current study were clinically healthy. More recently, Li et al. (2022) fed weanling pigs either a basal control diet or the basal diet supplemented with 0.10% GML and observed increased IL-10 and reduced TNF-α, both indicative of enhanced immune function. Pigs used in the current study were from a facility with no known pathogen challenges, which would potentially explain the lack of response seen in serum parameters due to dietary treatment. However, the increased circulating TNF-α observed in all pigs from days 0 to 14 may suggest an inflammatory response was taking place. Future work should evaluate immunoglobulin and pro-inflammatory cytokine levels at a more localized site, such as the small intestine, and perhaps quantify gene expression to gain a more functional understanding of how these acidifiers impact immune status.

In summary, feeding a blend of formic acid and GML showed promise to improve weanling pig growth performance immediately post-weaning. However, the inclusion of GML alone reduced ADG and feed conversion for the entirety of the nursery period, resulting in lighter BW by the end of the experiment. We observed no evidence that feeding formic acid or GML alone or in combination impacted fecal microbial populations or immune status. Further investigation into the use of this acidifier blend to benefit growth performance, the microbiota of the GI tract, and mucosal immunity is warranted.
